# Highly Sensitive Electrochemical Sensor for Diagnosis of Diabetic Ketoacidosis (DKA) by Measuring Ketone Bodies in Urine

**DOI:** 10.3390/s21144902

**Published:** 2021-07-19

**Authors:** Anna Go, Sung Ryul Park, Yejin Ku, Mingge Sun, Sangho Yeon, Jin-Kyun Lee, Sang Wook Lee, Min-Ho Lee

**Affiliations:** 1 School of Integrative Engineering, Chung-Ang University, 84 Heukseok-ro, Dongjak-gu, Seoul 06974, Korea; missanna01@cau.ac.kr (A.G.); stsrpark@cau.ac.kr (S.R.P.); mingge@cau.ac.kr (M.S.); yeonviolet@cau.ac.kr (S.Y.); 2 Program in Environmental and Polymer Engineering, Department of Polymer Science and Engineering, Inha University, 100 Inha-Ro, Nam-Gu, Incheon 22212, Korea; dkdlehdcns22@naver.com (Y.K.); jkl36@inha.ac.kr (J.-K.L.); 3 Bio-Health Product Research Center, Inje University, Gimhae-si 50834, Korea; 4 PCL Inc., Star Valley, 99, Digital-ro-9-gil, Geumcheon-gu, Seoul 08510, Korea

**Keywords:** ketone body, electrochemical sensor, cyclic voltammetry, non-invasive detection, point of care technology

## Abstract

In this report, we present an enzyme deposited Au electrode for an electrochemical measurement of acetylacetic acid (AcAc) in urine. The electrode has an immobilized layer of a mixture of D-β-hydroxybutyrate dehydrogenase (HBDH) and nicotinamide adenine dinucleotide (NADH) as sensing material to investigate its electroanalytical properties by means of cyclic voltammetry (CV). The modified electrodes are used for the detection of AcAc and present a linear current increase when the AcAc concentration increases. The electrode presents a limit of detection (LOD) of 6.25 mg/dL in the range of 6.25–100 mg/dL for investigation of clinical relevance. Finally, the electrode was evaluated using 20 patient samples. The measured results of urine ketone by the developed electrode were compared with the clinical results from a commercial kit, and the analysis showed good agreement. The proposed electrode was demonstrated to be a very promising platform as a miniaturized electrochemical analyzer for point-of-care monitoring of the critical biochemical parameters such as urine ketone.

## 1. Introduction

Electrochemical biosensor has substantial attentions in terms of their superior sensitivity, quick response time, as well as their potential application of rapid online analysis [1,2,3,4]. They can analyze a wide range of analyte molecules that can be detected easily using the surface modification process according to the analyte of interest [5,6]. Since an oxygen electrode was first developed for detection of glucose [7], many efforts have been performed to develop different types of biosensors in order to diagnosis disease [8,9]. Specifically, electrochemical biosensors for monitoring glucose and ketone bodies have been developed as a purpose of managing the diabetic patients [10,11].

Diabetic ketoacidosis, also known as DKA, is common in acute complications of diabetes and could be life threatening. In DKA, 3-β-hydroxybutyrate (3β-HB) is accumulated in blood and converts to acetone acetate (AcAc) by the action of 3-hydroxybutyrate dehydrogenase (3HBDH). At the same time, NAD+ was oxidized into NADH. Thus, serum 3β-HB concentration can be a biomarker reflecting the metabolic status in DKA [12]. For example, the concentrations of 3β-HB in blood are defined between 1 and 3 mM in hyperglycemia and above 3 mM in diabetic ketoacidosis (DKA), whereas 3β-HB below 1 mM recognized as healthy individuals [13]. Electrochemical biosensors for determination of 3β-HB in serum have been reported recently such as 3 HBDH immobilized coenzyme modified carbon nanotube-based electrodes [14], enzyme-based Clark electrodes [15], a screen printed fabricated with iridium carbon particle [16], etc. [17]. Nevertheless, sample collection from serum or blood is invasive, so it is painful, time consuming and may lead to trypanophobia.

Despite invasive methods such as finger pricking, a non-invasive one can provide simple and non-painful sample collection [18,19,20]. Therefore, a non-invasive urine ketone test can be an alternative and used as an early screening of diabetic ketoacidosis (DKA) patients. Even though urine ketone is a relatively poor diagnosis marker of DKA compared with blood ketone, the obvious advantage of using urine instead of blood for analysis of ketosis is the non-invasive nature of urine. The criteria of DKA measurement in urine are serum bicarbonate ≤ 18 mmol/L, pH ≤ 7.3, anion gap ≥ 10 mmol/L, plasma glucose > 13.9 mmol/L and the expression of ketonuria, which provides a criterion for indirectly measuring the expression of DKA in blood. Handheld ketone-meters based on enzymatic detection are also available commercially [13]. However, most of commercialized enzymatic detection kits are formed in strip type, which cannot be reused, and hence the method becomes expensive. Thus, rapid and repeatedly usable ketone sensor gains public interests.

Since urine has an abundance of AcAc and absence of 3β-HB, the developed sensor should be targeting AcAc measurement. Here, we developed the multi-layer enzyme modified electrode for measuring ketone body in urinary solution. The enzyme layers consist of a mixture of 3HBDH and NADH for catalyzing AcAc into 3HB and NAD+. At the same time, some of reaction product of 3HB and NAD+ will return to AcAc and NADH by action of 3HBDH on the layer. The cyclic voltammetric measurement of AcAc solution were firstly performed using the electrode as a proof of principle. The electrode depicts its sensitivity at 6.27 mg/dL. It is also shown excellent reproducibility of the test based on 10 repetition tests, which present only 5% error rate. To show the clinical relevance of the electrode, 20 patient samples were collected and tested with CV measurement. We finally compared the result with conventional urine dipstick methods to evaluate the performance of the electrochemical biosensor. The results of the developed electrode show good correlation with conventional method and presents more accurate quantification. Moreover, the sensor allows to re-use more than 10 times, which can offer inexpensive but accurate method.

## 2. Materials and Methods

Materials and Reagents: All reagents were treated with ultrapure deionized water using the Milli-Q system. β-nicotinamide adenine dinucleotide sodium salt (NAD, N0632), Albumin from bovine serum (BSA, A7030), β-nicotinamide adenine dinucleotide hydrate (NADH, H7004), sodium 3-hydroxybutyrate (3β-HB, 54965), and glutaraldehyde solution (GA, G6403) were all purchased from Sigma Aldrich. Acetoacetate was standard sample of Acetoacetate Colorimetric Assay Kit (MAK199, Sigma Aldrich). Phosphate buffered saline pH 7.4 (PBS, 10010) was purchased from Gibco. 99.5% Isopropyl alcohol (IPA, 5035-4410) was purchased from DaeJung Chemicals & Metals. Additionally, 99.5% acetone was purchased in Duksan.

Urine sample: All samples are collected and anonymized according to the selection criteria that are scheduled to be discarded after treatment by a patient who visited the Catholic University of Korea Seoul ST. MARY’s Hospital. Dispose of safely: (1) Dispose of solid and liquid wastes used in the experiment by autoclaving at 121 °C for 15 min or longer. (2) When the specimen is discarded, the destruction of personal information is completely destroyed (incineration, shredding) in a way that cannot be restored, or a dedicated collection equipment is used, and it is taken in a way that cannot be restored. (3) The procedure of reviewing the suitability of specimens, collecting, preserving, managing, providing and disposing of specimens, etc., shall be managed in accordance with the Catholic University of Korea Seoul ST. MARY’s Hospital, Diagnostic Laboratory Medicine and Sample Management Regulations

**Figure 1:**
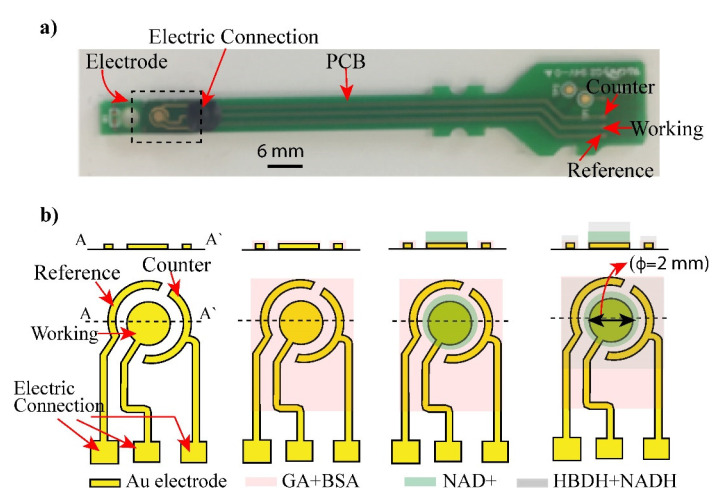
Layout of biosensor: (**a**) an electrode and its connected PCB was displayed. (**b**) Shape and structure of the electrode (Working, Counter and reference electrode) was illustrated. The electrode was coated with glutaraldehyde (GA) and bovine serum albumin (BSA) to form crosslinking with enzymes. Layers of NAD+ and mixture of HBDH and NADH were deposited on the electrode surface and addition of AcAc caused enzyme reaction.

Fabrication of electrode: Figure 1b shows the Au electrode structure consisting of working (WE), reference (RE), and counter electrodes (CE), which were fabricated by a conventional semiconducting process [20]. The electrode was fabricated from Cr/Au (50/100 nm) layers deposited on a glass substrate and patterned via the sequential etching of the chrome and gold layers. Cr was coated on Au to increase the adhesion of gold and glass substrate. After depositing chromium and gold layers, the electrode structure was patterned using standard photolithography and etched to obtain the shaped of three electrodes as in Figure 1.

The surface of electrode was cleaned by an acetone solvent and an isopropanol ethyl alcohol prior to coating with the reaction layers to remove pre-existed dirt, organic, and inorganic materials from the surface.

Enzyme deposition on the electrodes by deep coating method: After pre-cleaning of the electrode surface, immobilization of enzyme layers was performed on the surface. First, each chip was cleaned with oxygen plasma and then treated with glutaraldehyde (GA) and bovine serum albumin (BSA) to form crosslinking with enzymes. Next, 2 µL of NHD+ solution was dropped and casted on the electrode and dried out 37 °C for 30 min. To prepare an enzyme layer, a D-β-hydroxybutyrate dehydrogenase (HBDH) and nicotinamide adenine dinucleotide (NADH) at ratio of 1:1 were mixed. The mixture then was prepared by dispensing of solution (drop-cast) onto the working electrode. The coated electrodes were dried at incubator for 30 min. The modified electrodes were rinsed with deionized water and dried for 5 min at room temperature.

**Figure 2:**
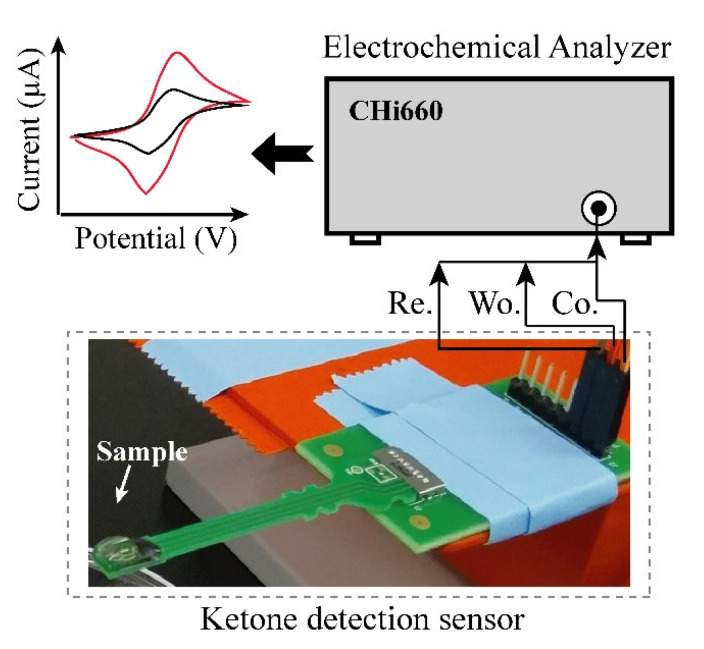
Layout of experimental set-up. Urine sample was dispensed on the electrode and cyclic voltammetry measuring (CV) was performed by connected CHI 660E.

Electrochemical experiment: Figure 2 shows the setup of the electrochemical experiment. AcAc in solution, which has 5 different concentrations (6.25, 12.5, 25, 50 and 100 mg/dL) was prepared for evaluation of developed urine ketone sensor. The buffer solution, which contained 20 mg/dL of uric acid, 20 mg/dL of ascorbic acid, and 4 mg/dL of acetaminophen, was also prepared for the measurement in the presence of interferent materials [21]. Cyclic voltammetry (CV) based measurements were performed using a CHI 600E (CH Instruments, Inc., Austin, TX, USA) potentiostat equipment for the quantification of AcAc in the solutions. CV was carried out with a potential range from −0.4 V to 0.4 V with scan rate of 100 mV/s. To show the clinical relevance, we tested the performance of the sensor using real urine samples, which were collected from healthy and patient individuals at Korea University Hospital of Guro, Seoul, South Korea, for the performance test using the fabricated electrode.

## 3. Results and Discussion

Electrochemical biosensors for AcAc detection have been developed in terms of their superior sensitivity, quick response time, as well as the wide range of analyte molecules that can be detected easily using the surface modification process according to the analyte of interest. Since urine has an abundance of AcAc and absence of 3β-HB, the developed sensor should be targeting AcAc measurement. The developed electrode has enzyme multilayers, which are composed of first an NAD+ layer and a mixture of HBDH and NADH. Using the developed electrode, AcAc can be converted into 3β-HB and NAD+ by enzyme reaction of HBDH and NADH, and then the produced 3β-HB returns to AcAc and NADH by action of HBDH and NAD+. During this process, the electrode can measure this reduction reaction by cyclic voltammetry.

**Figure 3:**
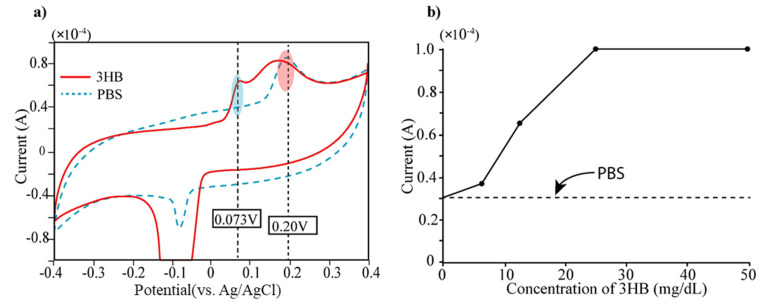
(**a**) Measurement of current level of 3-HB (mixed with PBS) and PBS using cyclic voltammetry (CV). Two current peaks appear in current level of 3-HB solution both 0.073 V and 0.25 V. Since PBS has single current peak at 0.25 V, the current peak at 0.073 V can be generated by 3HB. (**b**) The 3-HB solutions with different concentrations (0, 6, 12.5, 25, 50 mg/dL) were prepared by dilution using PBS and performed CV measurement. The current peaks of five different concentrations of 3-HB at 0.073 V were displayed.

3β-HB detection: The bare electrode was firstly tested to determine 3β-HB, which is the reaction product of the acetoacetate acids. At first, we measured cyclic voltammetry of 3β-HB (12.5 mg/dL) and displayed the result in Figure 3. We also measured PBS solution (Phosphate-Buffered Saline, pH 7.0) because PBS was used as buffer solution for diluents of 3β-HB. The cyclic voltammetric measurement was carried out with a potential range of −0.4~0.4 V with scan rate of 100 mV/s. The Au electrode presents two oxidation peaks at 0.073 V and 0.25 V, respectively (see Figure 3a). Taking into consideration that bare electrodes do not present any defined peak within the electrochemical window, we can determine the current peak at 0.25 V as arising from PBS buffer. Secondly, the 3β-HB in solution that has five different concentrations (0, 6.25, 12.5, 25, 50 mg/dL) were prepared and placed on the electrode for measurement of electric currents by cyclic voltammetry (CV). In Figure 3b, the current level of each concentration at the applied potential of 0.073 V was displayed. It is obvious that the current peak at 0.073 V increased according to the concentration of 3β-HB (see Figure 3b) and was saturated at the concentration of over 30 mg/dL of 3β-HB.

**Figure 4:**
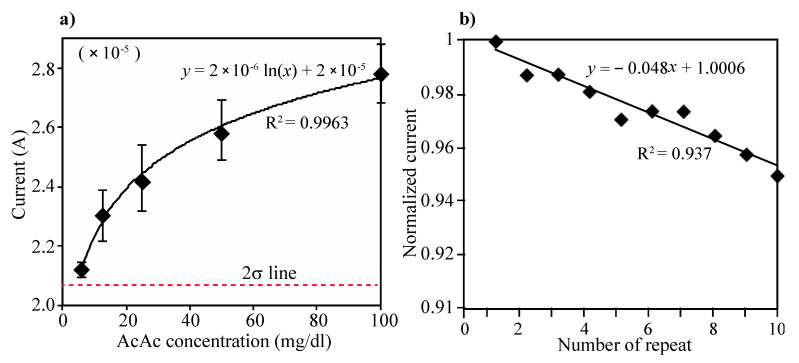
(**a**) Changes of current level according to the concentration of AcAc. Five different concentrations of AcAc (6.25, 12.5, 25, 50 and 100 mg/dL) and PBS (0 mg/dL) were prepared and performed CV measurement. Then, 80 µL of each prepared sample were added on the electrode and the current was measured at the applied potential of 0.073 V. The limit of detection (LOD) was defined as the lowest detectable signal from 2-sigma standard deviation above the mean current value at 0 mg/dL of AcAc solution. The enzyme-modified electrode can detect AcAc down to 6.25 mg/dL (sensitivity) and up to 100 mg/dL, (**b**) Ten repeated CV measurements and washing test were performed using 50 mg/dL of AcAc solution. All the measured current levels were normalized by first measurement.

Acetoacetate acid (AcAc) detection: The electrode consists of two enzyme layers, NAD+ and mixture of HBDH and NADH. The layers can generate two steps of reaction during the electrical measurement, which first the AcAc can transform into 3-HB by action of HBDH and NADH on the immobilized layer and then produced 3-HB from the first reaction can return to AcAc by action of HBDH and NAD+ on the electrode. Different concentration of AcAc solution (0, 6.25, 12.5, 25, 50 and 100 mg/dL) were prepared and tested using the developed urine ketone sensor. Then, 80 µL of each prepared sample were added on the electrode (see Figure 2) and the current was measured at the applied potential of 0.073 V. Three repeated tests were performed in each concentration of AcAc. As in Figure 4a, the current levels increased as AcAc concentration increased, and the current values were compared that of 0 concentration of AcAc. The limit of detection (LOD) was calculated as the lowest detectable concentration that produced a display value two standard deviations above the mean of the value displayed for a blank sample (0 mg/dL of AcAc). The enzyme-modified electrode can detect AcAc down to 6.25 mg/dL (sensitivity) and up to 100 mg/dL. The variations of coefficient (C.V.) in currents were measured to be within C.V. of 6% in the range of 0–100 mg/dL (see Table 1).

Repeated test: To evaluate reproducibility of the electrode, we performed 10 repeated measurements and washings for measuring 50 mg/dL of AcAc solution. As can be seen in Figure 4b, all the measured current levels were normalized by first measurement. Although the signal intensity has been gradually decreased (less than 5%) as times of measurements increase, the performing ability appeared to be maintained even over 10 times of repeated measurements.

**Figure 5:**
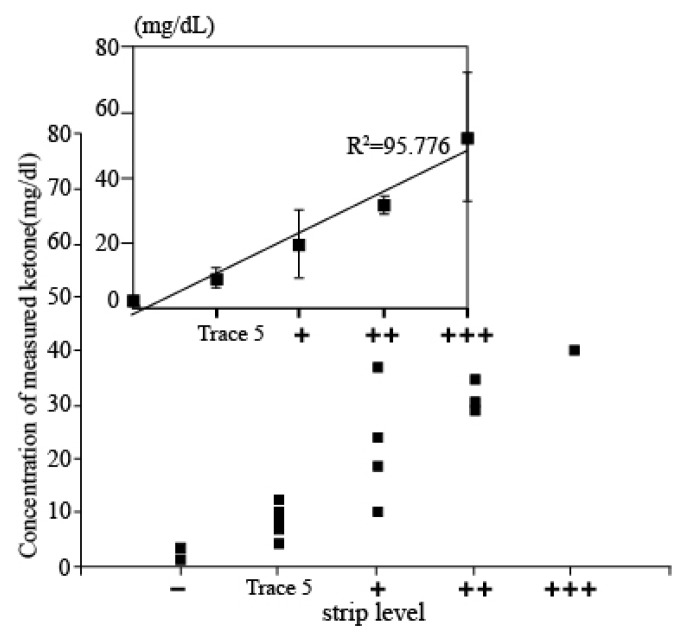
Associated plot between the electrochemical sensor and the dipstick methods acquired from 20 patients. *x*-axis presents ketone level detected by strip and *y*-axis shows ketone level measured by developed electrochemical sensor.

Evaluation using Clinical sample: In order to assess the accuracy of the measured results of the proposed electrochemical sensor, the results obtained from the sensor and the conventional dipstick measurement were compared by investigating the correlation between the measured data of both methods of those patients who were diagnosed with DKA. Then, 20 urine samples were firstly tested by dipstick methods and classified into five groups depending on the concentration of ketone (+++: up to 100 mg/dL; ++: up to 50 mg/dL; +: up to 10 mg/dL; trace 5: 10 mg/mL, and – is normal level). Then, 80 µL of urine samples were dispensed on the electrode and we performed the electrochemical measurements. The current values from the measurement were derived as concentration values using a current-concentration conversion formula in urine. Figure 5 shows the associated plot between the electrochemical sensor and the dipstick methods acquired from 20 patients. The *x*-axis shows the five different concentration levels of strip tests, and the *y*-axis shows the concentrations of AcAc using the developed ketone sensor. It can be seen that the measurement concentration is increasing according to the dipstick level. In addition, when compared with results from dipsticks, both results were highly correlated, demonstrating the clinical feasibility of our developed ketone sensor.

## 4. Conclusions

This report presents our custom-built Au electrode that can be installed into the portable reader device for the purpose of an accurate and rapid determination of ketone levels in urine. Electrochemical biosensors for serum or blood ketone measurement have been developed so far however it could be first attempt to develop electrochemical biosensor for detecting ketone in urine. The electrode consists of two enzyme layers, NAD+ and mixture of HBDH and NADH. The layers can generate two steps of reaction during the electrical measurement, which first the AcAc can transform into 3-HB by action of HBDH and NADH on the immobilized layer, and then 3-HB produced from the first reaction can return to AcAc by action of HBDH and NAD+ on the electrode. Our electrode can detect AcAc level from 6.25 mg/dL to 100 mg/dL with excellent linearity (R^2^ = 99.63 of log scale).

To evaluate clinical relevance, we collected 20 clinical samples of urine and measured the level of ketone using our developed biosensor and convectional dipstick measurement units. Both have over 95% correlation, which shows the feasibility of clinical usage of our device. The developed sensor format displayed good linearity as well as stability in ketone quantifying tests. The NADH and HBDH multi-layered sensor format is capable of providing digitally quantifiable concentrations of ketone bodies present in urine samples, which is comparable to the currently used dipstick urine ketone test kit that only shows the qualitative results. The performance of our sensor could be further implemented with a read-out device. Therefore, our future work will be an integration of this reusable ketone sensor with a portable device as a clinical test.

## Figures and Tables

**Table 1:** Coefficient of variance (C.V.) results of different AcAc concentration at three repetition tests.

Concentration (mg/dL)	C.V. (%)
0	6
6.25	1
12.5	4
25	5
50	4
100	4

C.V.: coefficient of variance.
